# Case-Based Virtual Reality Simulation for Severe Pelvic Trauma Clinical Skill Training in Medical Students: Design and Pilot Study

**DOI:** 10.2196/59850

**Published:** 2025-01-17

**Authors:** Peng Teng, Youran Xu, Kaoliang Qian, Ming Lu, Jun Hu

**Affiliations:** 1 Department of Teaching Resources Management Teaching Management Office of Nanjing Medical University Nanjing China; 2 School of Stomatology Nanjing Medical University Nanjing China; 3 Department of Orthopedics First Affiliated Hospital of Nanjing Medical University Nanjing China; 4 Department of Pharmacology & Jiangsu Key Laboratory of Neurodegeneration Nanjing Medical University Nanjing China

**Keywords:** case-based learning, virtual reality, pelvic fracture, severe pelvic trauma, hemodynamic instability, clinical skill training, VR, pelvic trauma, medical student, pilot study, orthopedic surgery, theoretical teaching, acceptability

## Abstract

**Background:**

Teaching severe pelvic trauma poses a significant challenge in orthopedic surgery education due to the necessity of both clinical reasoning and procedural operational skills for mastery. Traditional methods of instruction, including theoretical teaching and mannequin practice, face limitations due to the complexity, the unpredictability of treatment scenarios, the scarcity of typical cases, and the abstract nature of traditional teaching, all of which impede students’ knowledge acquisition.

**Objective:**

This study aims to introduce a novel experimental teaching methodology for severe pelvic trauma, integrating virtual reality (VR) technology as a potent adjunct to existing teaching practices. It evaluates the acceptability, perceived ease of use, and perceived usefulness among users and investigates its impact on knowledge, skills, and confidence in managing severe pelvic trauma before and after engaging with the software.

**Methods:**

A self-designed questionnaire was distributed to 40 students, and qualitative interviews were conducted with 10 teachers to assess the applicability and acceptability. A 1-group pretest-posttest design was used to evaluate learning outcomes across various domains, including diagnosis and treatment, preliminary diagnosis, disease treatment sequencing, emergency management of hemorrhagic shock, and external fixation of pelvic fractures.

**Results:**

A total of 40 students underwent training, with 95% (n=38) affirming that the software effectively simulated real-patient scenarios. All participants (n=40, 100%) reported that completing the simulation necessitated making the same decisions as doctors in real life and found the VR simulation interesting and useful. Teacher interviews revealed that 90% (9/10) recognized the VR simulation’s ability to replicate complex clinical cases, resulting in enhanced training effectiveness. Notably, there was a significant improvement in the overall scores for managing hemorrhagic shock (t_39_=37.6; 95% CI 43.6-48.6; *P*<.001) and performing external fixation of pelvic fractures (t_39_=24.1; 95% CI 53.4-63.3; *P*<.001) from pre- to postsimulation.

**Conclusions:**

The introduced case-based VR simulation of skill-training methodology positively influences medical students’ clinical reasoning, operative skills, and self-confidence. It offers an efficient strategy for conserving resources while providing quality education for both educators and learners.

## Introduction

Severe pelvic trauma is characterized by unstable pelvic fractures resulting from high-energy impacts, typically accompanied by complications such as fatal massive bleeding and organ injuries. The mortality rate in China is as high as 10%-30% [1-3]. Managing pelvic fractures with hemodynamic instability poses a significant challenge within the orthopedic surgery discipline [4,5]. The current gap that exists in the field of the effectiveness of severe pelvic trauma clinical skill training is multifaceted. It includes the inaccessibility of clinical teaching in hospitals and the constraints of traditional classroom instruction. In addition, owing to the complexity, unpredictability of treatment locations, the rarity of typical cases, reluctance to cooperate, and ethical concerns surrounding clinical teaching, traditional methods of clinical skill training in diagnosing and treating severe pelvic trauma are limited to theoretical instruction and mannequin-based simulation. These methods face limitations, including disproportionate teacher-student ratios, high model consumption, insufficient training spaces, the absence of comprehensive and immediate feedback, and inadequate training overall. Specifically, on-site mentoring can be challenging to achieve effectively and efficiently. Therefore, a teaching model for severe pelvic trauma needs to integrate new teaching strategies and computer technology to address these issues. This study addresses gaps in the current training methods and tools for managing severe pelvic trauma by designing an innovative virtual reality (VR)–based simulation platform.

In recent years, VR technology has gained widespread acceptance in orthopedic surgery education because of its multisensory immersive experience, the convenience of real-time interaction, and a psychologically secure experimental environment [6,7]. These technologies have mitigated the limitations of time, space, and teaching resources in orthopedic surgery education and addressed issues such as simulated patients and the difficulty of repeated practice. To some extent, this has enhanced the efficiency and quality of clinical practice. Digital patient simulators have become valuable tools in medical education, offering a standardized method of patient simulation [8,9]. However, virtual patients typically only exhibit clinical symptoms and signs, with minimal explanation of the underlying fundamental medical knowledge of primary symptoms and positive or negative signs. Furthermore, there is a scarcity of research on virtual simulation teaching models that integrate basic and clinical medicine for severe pelvic trauma. To mimic the real teaching scenario of the disease and capture the sudden and variable condition of patients in clinical settings, we developed an electronic standardized patient (ESP) for severe pelvic trauma, capable of replicating both macroscopic changes, such as monitor display data, and microscopic changes, including alterations in blood circulation, organs, tissues, and cells. The ESP model facilitates the integration and application of clinical and basic knowledge. The original design and technology of the ESP were conceived by Professor Xing Ya Gao’s team at Nanjing Medical University, Department of Physiology [10]. An ESP is grounded in contemporary theories of human systems physiology and incorporates relevant clinical literature and data through analog circuits, physics, and other methods to formulate a mathematical model. Artificial intelligence and data analytics are used to refine and adjust the simulation data. In summary, the ESP represents a web-based intelligent standardized patient, enabling students to interact with it in a virtual hospital setting from a physician’s perspective. To date, the ESP has not been fully used in the clinical skills education for severe pelvic trauma.

Case-based learning (CBL) is recognized as an effective teaching strategy in clinical skills training [11,12]. CBL is particularly valued in orthopedic surgery education for its ability to enhance learner participation, foster active learning, and develop critical thinking and problem-solving skills [13]. CBL is an educational method designed to analyze medical records, recreate real clinical scenarios, and engage learners in addressing actual clinical challenges, thereby stimulating their curiosity and promoting active learning [14].

CBL, combined with VR simulation technology, has been successfully applied in midwifery laboratory courses, with its effectiveness widely acknowledged by students [15]. However, the application of these combined methodologies in teaching clinical skills for severe pelvic trauma has yet to be explored. Therefore, in this study, we introduced a case-based digital skill training program for severe pelvic trauma and conducted mixed methods to evaluate its acceptance among users. Additionally, we implemented a pretest-posttest design to investigate the potential impact of this clinical skills training on undergraduate and graduate students. We hypothesize that the case-based VR simulation teaching method will effectively complement current training practices for severe pelvic trauma, enhancing knowledge, procedural skills, and confidence, while also improving instructional efficiency and effectiveness for educators.

## Methods

### Study Design

**Figure 1:**
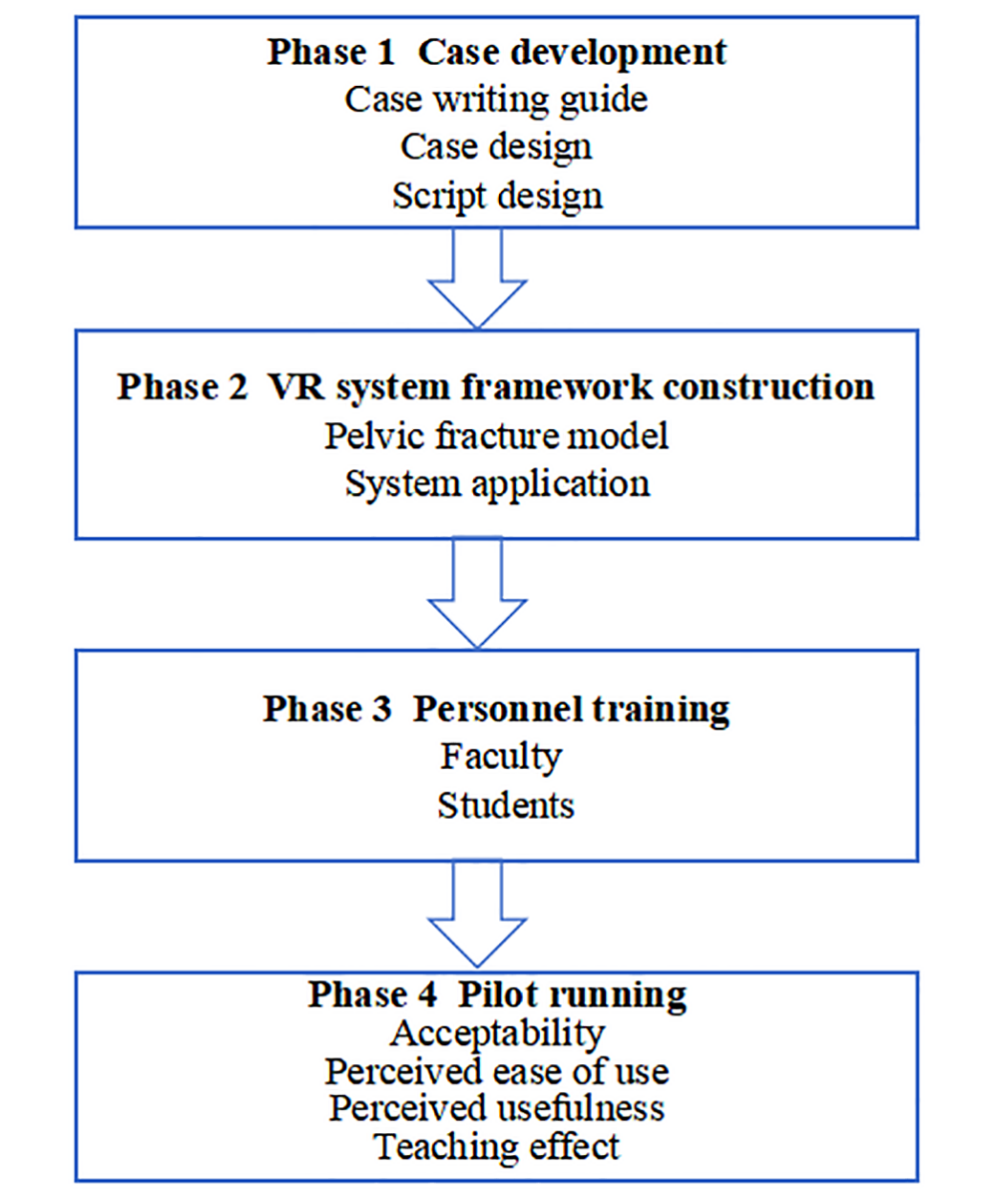
A flow diagram illustrating the steps involved in developing a case-based VR simulation for severe pelvic trauma clinical skill training. VR: virtual reality.

We conducted a 4-phase study (Figure 1). First, we created the simulated teaching case base and adapted typical clinical cases based on the case writing guide [16,17]; subsequently, the case script was developed. Next, we established the framework of the VR system, comprising 3 ports and 3 system modules. To facilitate the effective implementation of the teaching system, we formulated a comprehensive training plan for department administrators, course instructors, and medical students. A pilot test of the system was conducted on a limited scale, followed by a 1-group pretest-posttest design to assess its acceptability, potential application, and existing limitations.

### Phase 1: Case Development

#### Case Design

**Figure 2:**
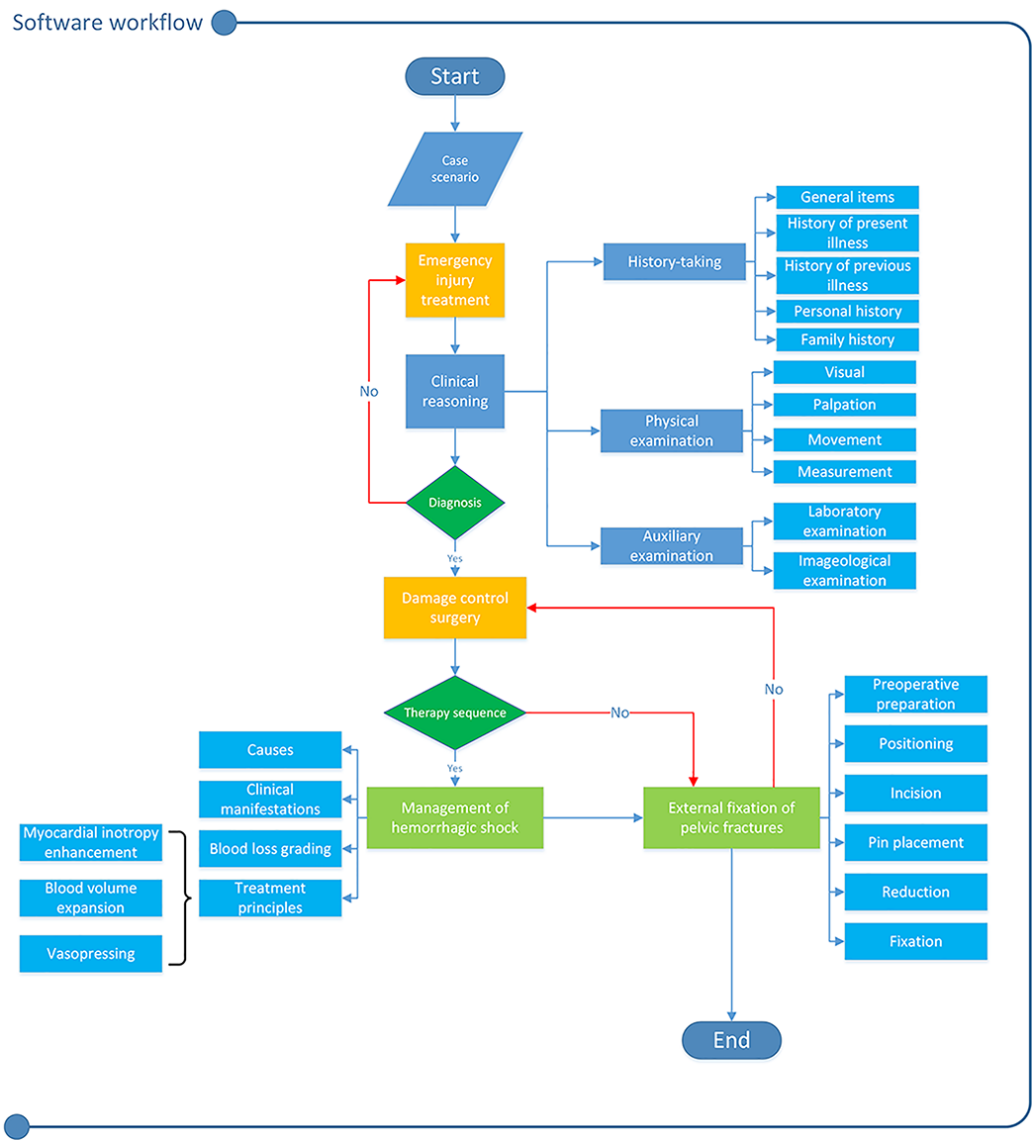
An optimal template for the diagnosis and treatment process of severe pelvic trauma (blue arrows for the correct path, red arrows for the wrong path).

Specialists in basic and clinical medicine, drawing on a literature review, real patient cases, and the course syllabus, designed the cases. A representative case involved a worker who fell from a high platform and was sequentially evaluated in the emergency department, admitted to the intensive care unit, and taken to the operating room as his condition worsened. The diagnostic and treatment processes for this condition were standardized. Learners were guided through different scenarios to acquire both declarative and procedural knowledge for managing patients with severe pelvic trauma. We developed 3 initial cases, each representing a different stage of trauma and treatment approach (Table 1). Learning paths for diagnosis and treatment were outlined in a flowchart (Figure 2), based on clinical treatment guidelines [18,19] to serve as a framework for evaluating student performance. We collected clinical manifestations, relevant imaging, and laboratory examination results of real patients.

**Table 1:** Three cases with patients diagnosed with pelvic trauma.

	Case 1	Case 2	Case 3
Diagnosis	Pelvic fracture	Pelvic fracture with mild hemorrhagic shock	Pelvic fracture with severe hemorrhagic shock
Injury evaluation	Mild	Moderate	Severe
Diagnostics	Standard	Plus computed tomography of the pelvis	Plus computed tomography of the pelvis and abdomen
Therapy	Surgery	Antihemorrhagic shock therapy, surgery	Antihemorrhagic shock therapy, surgery

The 3 cases varied in the severity of pelvic trauma, necessitating different diagnostic and treatment strategies.

#### Script Design

**Figure 3:**
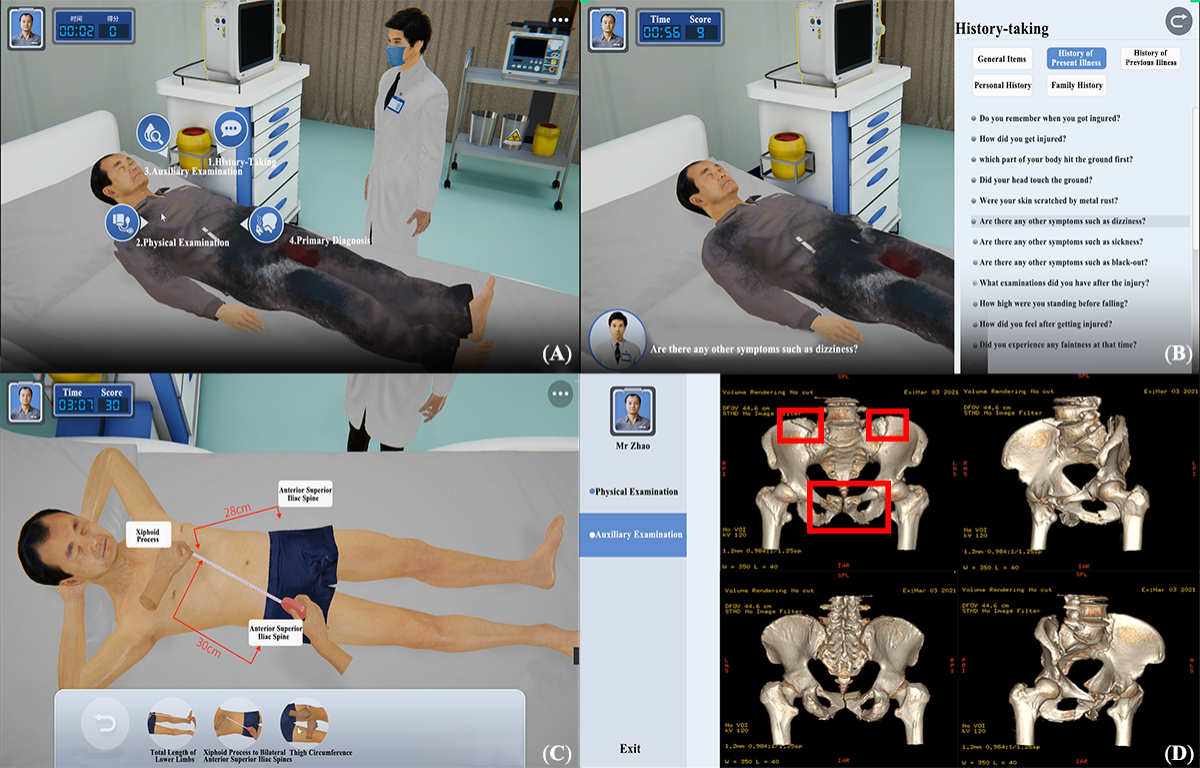
Screenshot of the emergency room. (A) The user acts as a doctor in the emergency room while interacting with the ESP by selecting different diagnoses and treatment icons. (B) Trainees can choose the present history module in history taking to communicate with the ESP. (C) During the physical examination session, students can use a tape measure to measure the distance between the bilateral anterior superior iliac spine and the xiphoid process to determine whether the pelvis was displaced. (D) The students order a computed tomography scan to evaluate the location and severity of the ESP’s injury. ESP: electronic standardized patient. Please note that a higher resolution version of this image can be found in Multimedia Appendix 1.

Scripts focused on scenarios in emergency rooms, intensive care units, and operating rooms. In a virtual emergency room, users could interact with the ESP, using various diagnostic and treatment methods, such as history taking, physical examination, and auxiliary examination, to formulate a preliminary diagnosis based on evidence gathered during the process. Learners engaged with the ESP through text input or voice chat during the history-taking session. They then entered pertinent information into the system’s modules for general items, history of present illness, personal history, marital history, and family history. The system evaluated 4 aspects: completeness of the inquiry framework, logical order of inquiries, communication skills, and awareness of humanistic care, assigning scores based on the accuracy of the information collected. The system also recorded the total duration of inquiries and the time spent on each module for later analysis. Physical examination, a fundamental skill for diagnosing diseases, involves comparing and identifying positive signs. The system assessed the patient examination position, completeness and sequence of operational steps, standardization of procedures, comprehensiveness of examination content, accurate reporting of results, and basic professional quality. Auxiliary examination, crucial for diagnosis and treatment planning, involves evaluating laboratory tests and computed tomography images. Upon completing these steps, students made a preliminary diagnosis and a prioritized list of differential diagnoses. Correct diagnoses led to immediate treatment initiation; otherwise, students continued trial and error in this module (Figure 3).

**Figure 4:**
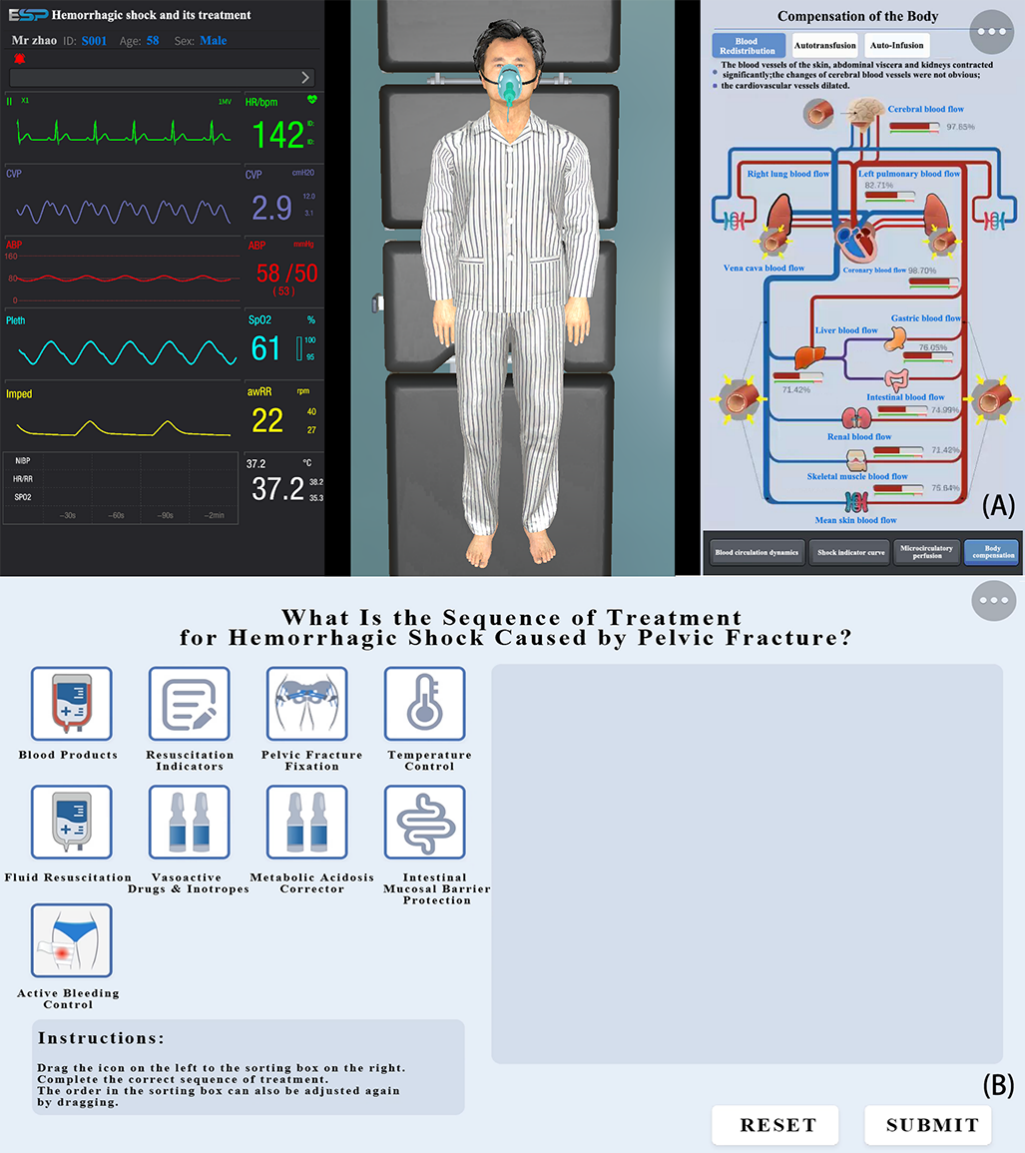
Screenshot of hemorrhagic shock and its treatment. (A) Monitors in the intensive care unit show decreased blood pressure, increased heart rate, and subnormal central venous pressure and arterial oxygen saturation in the ESP. By users’ observation, the ESP at this time showed pallor, decreased urine output, and cold sweats. Users can also learn about the compensatory mechanisms of ESP in terms of blood redistribution, autotransfusion, and auto-infusion at the system, organ, and tissue levels after hemorrhagic shock. Screenshot of treatment principles for severe pelvic trauma. (B) In this module, the user should drag the corresponding icons from all the treatment measures given on the left to the right blank according to the treatment principle. When the dragged content matches the built-in answer of the system, the next section will be entered. ESP: electronic standardized patient. Please note that a higher resolution version of this image can be found in Multimedia Appendix 1.

Upon initiating appropriate immediate treatment measures, the ESP was transferred to the intensive care unit. Vital sign monitors reflected real-time changes based on the condition and treatment progression. In this module, users learned about the causes, clinical manifestations, severity classifications, and management of hemorrhagic shock (Figure 4).

**Figure 5:**
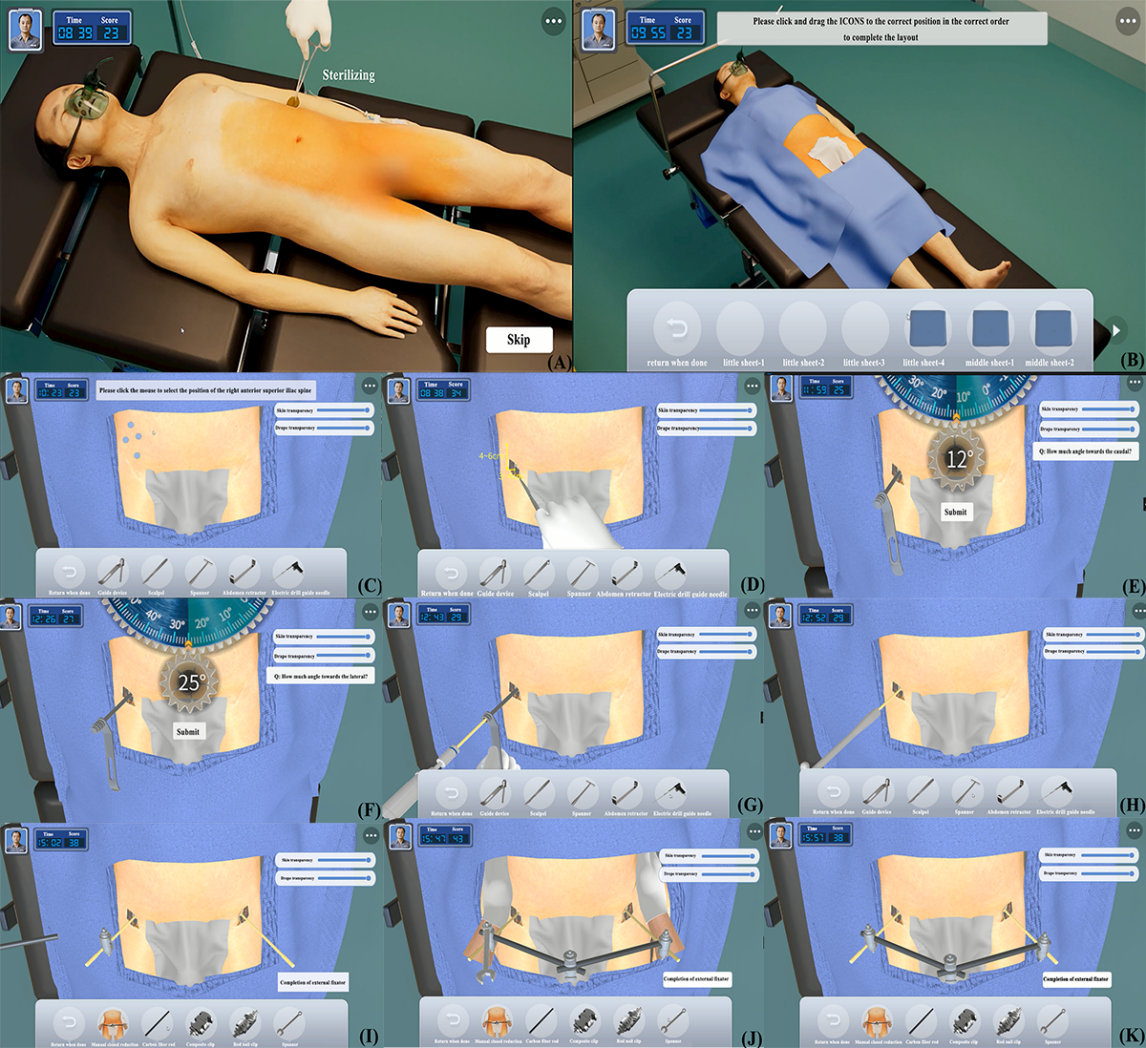
Screenshot of the surgical procedure of external fixation of pelvic fractures. (A) According to the sequence of operation, the users need to complete disinfection, (B) laying, (C) positioning, (D) incising, (E,F) positioning and protection of the guide, (G) electric insertion of screw, (H) manual insertion of screw, (I) use of rod-nail clamp and composite clamps, and (J,K) adjustment of the external fixator after manual reduction. Please note that a higher resolution version of this image can be found in Multimedia Appendix 1.

During the surgical procedure for external fixation of a pelvic fracture, users first completed preparatory tasks such as handwashing and donning surgical attire. Then, the ESP underwent anesthesia, sterilization, positioning, drilling, and bracket installation according to surgical standards (Figure 5).

### Phase 2: Framework Construction

#### Construction of Pelvic Fracture Model

To provide learners with a comprehensive understanding of pelvic anatomy and fracture morphology, we collaborated with software engineers to develop the relevant content. The development process involved several steps. Initially, 3D modeling data of pelvic fractures were extracted from real clinical cases, and a preliminary geometry was constructed using the Maya (Autodesk) development tool to complete the foundational model of pelvic fractures. Subsequently, the ZBrush (Pixologic) tool was used to sculpt and refine the fracture site shape, fracture line position, and bone surface structure of the basic pelvic fracture model, minimizing the detailed morphology of the pelvic fracture. Next, we used the support model subdivision level adjustment feature to enhance the pelvic fracture model’s subdivision level, showcasing the complex structure of the fracture site. The addition of materials and textures in Maya, such as bone texture and skin color and texture, further improved the visual realism. The established skeletal system was then integrated into the pelvic fracture model, and animations were created based on real pelvic fracture case data, including the degree of fracture displacement and the relative position of fracture blocks. Finally, the pelvic fracture model was exported from Maya to Unity (Unity Technologies) for digital and real-time rendering, allowing learners to interact with a pelvic fracture model in a virtual environment to simulate actual surgical procedures. The development and construction process of the pelvic fracture model is elaborated in Multimedia Appendix 2.

#### Construction of System Application

The digital simulation system was segmented into 3 ports: the department administrator, course leader, and user ports, constructed on a website platform. The department’s primary role is to create the appropriate courses in the system, either compulsory or optional. Once the courses are established, they should be linked to the digital simulation skills training system, and relevant instructors assigned to teach all enrolled students for the semester. Course instructors are responsible for preparing preview materials, learning videos, self-assessment questions, and postclass surveys. They also review experimental outcomes and evaluate their performance within the system. Users can engage with the digital simulation system for severe pelvic trauma by enrolling in or attending a scheduled course. Upon system login, the interface offers 2 modes: training and assessment. Due to the digital simulation experiment teaching integration with the virtual simulation experiment teaching sharing platform of Nanjing Medical University, campus users are not required to register or authenticate before use, thanks to unified identity verification and experimental data integration.

### Phase 3: Personnel Training

#### Faculty Training

Faculty training targeted course directors and instructors from various disciplines within basic and clinical medicine, aiming to familiarize them with the digital simulation experimental teaching system. They received training on case materials, experimental teaching objectives, principles, teaching processes and methods, steps, outcomes, and conclusions. Additionally, they learned to address technical system issues, respond to student inquiries, and interact with students on the platform during experiments.

#### Student Training

Before software utilization, students were informed about the operating system and hardware configuration requirements. The application runs on a Windows 7 64-bit or higher PC, equipped with a 3.60 GHz Intel i5 processor, 8 GB RAM, NVidia GTX 2060 graphic card, and a 1920×1080 display resolution. The application supports various browsers on different operating systems, such as Google Chrome, the 360 browser, and Firefox. After accessing a specific URL, users must install MengooLauncher, requiring less than 100 MB of plug-in capacity, as indicated. Before proceeding to the autonomous training or assessment interface, users familiarize themselves with the experimental teaching objectives and principles through introductory and instructional videos.

### Phase 4: Pilot Running Evaluation of Digital Simulation Software

#### Design

A self-controlled teaching comparison study was conducted at Nanjing Medical University, China, from October 2023 to January 2024, to examine the impact on knowledge, skills, and confidence before and after using virtual simulation experimental teaching software. All participants underwent a knowledge assessment of equal difficulty before and after system engagement.

In the initial design phase, the teaching and research teams sought input from the software development team and feedback from various users through internal reviews. A case-based VR simulation of severe pelvic trauma was tested by students majoring in clinical medicine, clinical teachers, and basic medicine instructors. We distributed the ESP digital simulation teaching web link via WeChat (Tencent) to pertinent users, soliciting face-to-face or written feedback on case and script design and software development, including aspects related to clinical and basic medicine education. Additionally, the teaching research group reviewed classical cases of severe pelvic trauma and questionnaire responses.

#### Sampling and Recruitment

The recruitment criteria were as follows: (1) students must have completed courses in diagnostics, internal medicine, and surgery; (2) they needed to have a laptop for the study; (3) they should not have participated in any form of digital simulation software training for clinical skills prior; and (4) they agreed to participate in the pilot study and signed an informed consent form. We invited a purposive sample of 20 fourth-year undergraduates and 20 first-year graduate students majoring in clinical medicine from the First Clinical Medical College of Nanjing Medical University to test the case-based VR simulation software. Based on sample size requirements previously reported in the literature for evaluating data collection materials, a minimum of 10 samples is necessary to ensure the adequacy and validity of the assessment instrument [20]. The evaluation sought feedback from a diverse group of users, including undergraduate and graduate students, as well as teachers with various professional titles. The qualitative study used a representative population most familiar with the study topic, comprising 5 orthopedic teaching teachers and 5 basic medicine teachers.

#### Data Collection

Participants were required to complete the training and assessment using the digital simulation software for severe pelvic trauma treatment. Our data collection involved a repeated measurement approach to assess knowledge test scores before and after the simulation. Feedback on the simulation teaching tools was collected through a single questionnaire. Participation in the survey was entirely voluntary, and students were informed that their decision to participate would not impact their academic standing. The survey distribution was conducted independently, with no direct or known ties between the distributors and the students, ensuring an unbiased and pressure-free environment for participants. Participants were recruited and invited to complete the survey through the WeChat platform using the Wenjuanxing applet. The survey was administered anonymously to encourage honest feedback on their experience with the VR simulation software.

#### Outcome Assessment

Although evaluation questionnaires are commonly used to compare learning tools, there is a dearth of validated tools for assessing the ESP digital simulation software as a learning instrument. Consequently, the questionnaire was adapted from a validated assessment tool in educational literature, offering a resource for future research on the perception in clinical medical professional education. The questionnaire comprised 15 Likert-scale statements (1=strongly disagree to 5=strongly agree), assessing accessibility and usability. The teaching team and subject experts reviewed the questionnaire. Cronbach α for the questionnaire was 0.85 (n=15). The questionnaire was adapted from a validated assessment tool widely used in educational literature for clinical medical professional education [21]. The instrument used for assessing system acceptability was based on a modified version of the Technology Acceptance Model questionnaire, with validation provided by Balki et al [22]. The Cronbach α coefficient, which reflects internal consistency, was calculated jointly for both the usability and acceptability surveys, yielding a consistent value for both aspects.

#### Data Analysis

Descriptive analysis was applied to the quantitative data obtained from the Likert scale. For qualitative data, which included responses to 7 open-ended questions, we used a validated content analysis method as described by Elo and Kyngäs [23]. All participant comments were transcribed and imported into Excel (Microsoft Corp) for coding. The content analysis process consists of several steps: (1) familiarizing oneself with the data and the hermeneutic spiral, (2) dividing up the text into meaning units and subsequently condensing those meaning units, (3) formulating codes, and (4) developing categories and themes [24]. Initially, the primary investigator analyzed the content, and the research team subsequently reviewed and discussed the codes to achieve consensus. In cases of disagreement, group discussions were held, and, if necessary, a third-party opinion was sought to ensure triangulation and enhance reliability. The 7 open-ended survey questions are provided in Multimedia Appendix 3 for reference.

For user acceptance analysis of the ESP platform among undergraduates, graduates, and tutors, descriptive statistics were used. To compare the mean rating scales of each survey item between groups, an independent 2-tailed *t* test was performed with a significance threshold of *P*<.05. Prior to conducting parametric tests, we verified the assumption of normality using the Shapiro-Wilk test, confirming that the data met the requirements for a parametric approach. This method was chosen over nonparametric tests due to the normal distribution of the data, making it suitable for our sample size and study design.

### Ethical Considerations

The Nanjing Medical University ethics committee approved this study (2023418). During the informed consent process, participants were made aware that no incentives were provided for participation in the survey. All methods were implemented in accordance with the Helsinki declaration. All participants were voluntary in the study.

## Results

### Demographic Results

Of the 56 students enrolled in the optional course on the integrated case of severe pelvic trauma in October 2023, 40 students consented to participate in the pilot study. Among these participants, 50% (n=20) of the students were senior-year undergraduates, 50% (n=20) of the students were first-year graduates (n=20), 45% (n=18) of students were men, and 55% (n=22) of the students were women. The mean age was 22.9 (SD 1.3) years. Among the undergraduate participants (n=20), there were 10 male and 10 female students, with a mean age of 21.9 (SD 0.9) years. For the graduate participants (n=20), there were 8 male and 12 female students, with a mean age of 24.0 (SD 0.8) years. Ten faculty members with at least 5 years of teaching experience in orthopedic surgery or basic medicine were also invited to participate. Neither the students nor the faculty had prior experience with this type of digital simulation platform. All participants were required to complete the questionnaire shortly after finishing the training tasks.

### Questionnaire Results

A 5-point Likert scale assessed perceptions of the acceptability, effectiveness, and applicability, summarized in Table 2.

**Table 2:** Average rating scores of survey questions (Q1-Q15) by all students.

Survey questions	Average rating scores, mean (SD)	
	Undergraduate students	Graduate students
(1) The digital software provides a simulation of a real patient	4.40 (0.66)^a^	4.80 (0.40)
(2) During the simulation, I felt like a doctor taking care of this patient	3.90 (0.99)	3.90 (1.14)
(3) When I finished the simulation, I felt I had to make the same decisions as doctors in real life	4.55 (0.50)	4.80 (0.40)
(4) The VR^b^ simulation is interesting and useful	4.85 (0.36)	4.70 (0.46)
(5) The difficulty of the VR simulation is appropriate to my own level of knowledge and skills	4.10 (0.94)^a^	4.60 (0.49)
(6) The feedback from the system adequately reflected my actual performance	4.80 (0.40)	4.60 (0.66)
(7) The goals of scenario simulation are clear and easy to understand	4.55 (0.59)	4.80 (0.40)
(8) I can access the system anytime and anywhere for simulation training	4.75 (0.54)	4.90 (0.30)
(9) The VR simulation can help me to use basic medical knowledge to explain clinical manifestations of clinical reasoning skills	4.80 (0.40)^c^	4.20 (0.87)
(10) The ESP^d^ simulator can help me develop clinical operation skills	4.30 (0.78)	4.60 (0.49)
(11) I feel more confident about working with hospital colleagues	4.40 (0.80)	4.70 (0.46)
(12) The VR simulation increased my confidence as a practicing physician	4.40 (0.66)	4.60 (0.49)
(13) The VR simulation can support courses and exams	4.60 (0.49)	4.75 (0.43)
(14) Compared with traditional teaching practice training methods, VR simulation can reduce my training cost and risk	4.90 (0.30)^c^	4.50 (0.50)
(15) In general, this VR simulation training should enhance my learning	4.80 (0.40)	4.80 (0.40)

^a^*P*<.05 compared to the graduate student group.

^b^VR: virtual reality.

^c^*P*<.01 compared with the graduate student group.

^d^ESP: electronic standardized patient.

**Figure 6:**
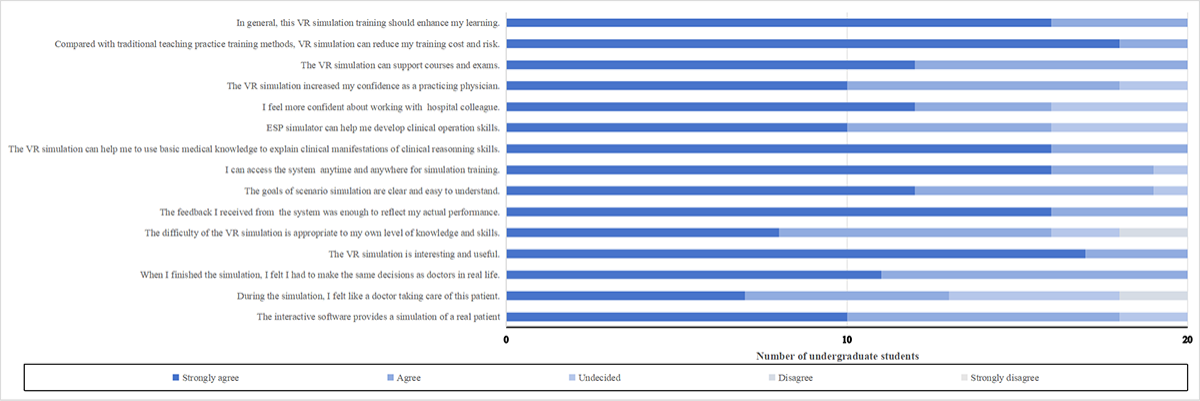
Acceptability, effectiveness, and applicability of the case-based VR software by undergraduate students. ESP: electronic standardized patient; VR: virtual reality. Please note that a higher resolution version of this image can be found in Multimedia Appendix 1.

**Figure 7:**
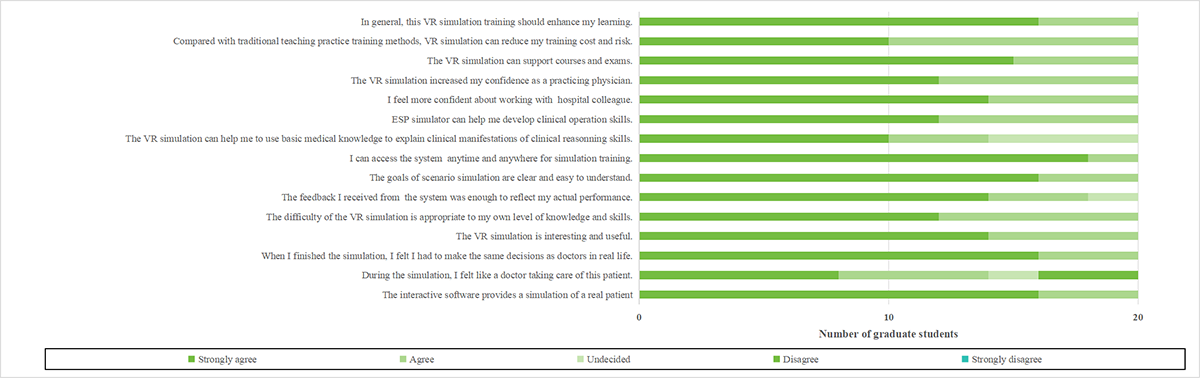
Acceptability, effectiveness, and applicability of the case-based VR software by graduate students. ESP: electronic standardized patient; VR: virtual reality. Please note that a higher resolution version of this image can be found in Multimedia Appendix 1.

The respondents showed strong agreement; 95% (n=38) agreed or strongly agreed that the interactive software simulated a real patient scenario (Q1 in Figures 6 and 7). However, 68% (n=27) agreed or strongly agreed that “During the simulation, I felt like a doctor caring for this patient” (Q2), with 18% (n=7) neutral and 15% (n=6) disagreeing. All students (n=40, 100%) felt they had to make real-life doctor decisions by the end of the simulation (Q3) and found the VR simulation interesting and useful (Q4).

Additionally, 90% (n=36) believed the VR simulation’s difficulty was appropriate for their knowledge and skills (Q5), while 5% (n=2) disagreed. Moreover, 95% (n=38) reported that the feedback from the system sufficiently reflected their performance (Q6). Most students (95%, n=38) understood the goals of the scenario simulation clearly (Q7). Nearly all students (98%, n=39) could access the system anytime for training (Q8), and 98% (n=39) agreed that “The VR simulation can help apply basic medical knowledge to clinical reasoning skills” (Q9).

When inquired if the ESP simulator aided in developing clinical operational skills (Q10), 85% (n=34) agreed. Regarding confidence in collaborating with hospital colleagues (Q11) and functioning as practicing physicians (Q12), 90% (n=36) agreed or strongly agreed. All students (n=40) concurred that the VR simulation supports courses and exams (Q13), is cost-effective compared to traditional training (Q14), and enhances learning overall (Q15).

### Impact of Training Level on Questionnaire Answers

Finally, a 2-tailed *t* test was used to compare the average rating scales between undergraduate and graduate students. This analysis aimed to determine if the responses varied according to their academic level. Specifically, for Questions 1 and 5, undergraduate students exhibited significantly stronger disagreement than their graduate counterparts, as indicated by the *P* values (Q1, *P*=.03; Q5, *P*=.047). Moreover, when compared to graduate students, a larger proportion of undergraduates believed that VR simulation could enhance their clinical reasoning abilities (Q9, *P=*.009) and decrease their training costs and associated risks (Q14, *P*=.004). Additionally, all participants indicated a low level of agreement with the statement “During the simulation, I felt like a doctor taking care of this patient” (Q2, *P*=.99). No significant differences were observed in responses to the remaining questions when analyzed based on academic level.

### Qualitative Analysis

#### Overview

The members of the research team conducted a 1-to-1 structured interview with the participating teachers around the interview outline of 7 open questions formulated in advance, as shown in Table 3.

**Table 3:** Themes of teacher groups’ interview on the application and research of digital simulation teaching curriculum system.

Theme	Teacher
Perceived benefits of systematic teaching	“As one of the most complex and urgent diseases in orthopedics, severe pelvic trauma often fails to receive on-site teaching from teachers in the tense emergency treatment site. In addition, due to the long treatment period of this disease, it takes a long time to fully learn the diagnosis and treatment process of this disease. However, in real life, learners only spend limited time rotating with one department and cannot follow through the entire disease process and treatment course. By creating typical cases of severe pelvic trauma and constructing a virtual clinical diagnosis and treatment environment based on ESP, this system shortens the learning cycle of students, enables learners to experience different treatment settings, allows a large scale concurrent online participation breaking through the limitations of traditional teaching in time and space and improving the efficiency of teaching organizations.”
The appropriateness of case application subjects	“It helps me conduct classified teaching according to the basic knowledge level of undergraduates and postgraduates. The basic medical and clinical medical knowledge involved in the disease set in the system is suitable for students at different undergraduate and postgraduate levels to learn. At the same time, the extensibility of the system enriches the flexibility and innovation of students’ training and assessment.”
The extendibility of course application	“The teaching design of this case is very suitable for the objective structured clinical examination scenario, which is closer to reality than traditional simulation training scenario. In addition, it introduces ESP, without the need for on-site re-placement of exam environments and standardized patient training.”
The limitations of systematic research	“In the early stage of communication and interaction with the ESP speech inquiry, it was found that the ESP lacked a large sample of language training model library, so it could not recognize the semantics of the trainer. In addition, the ESP has not achieved the language style characteristics of different types of characters at this stage.”
Recommendations for enhancing systematic research	“To enhance the virtual ESP simulation system, five improvements were suggested: enlarging the ESP case database, incorporating a feature for automatic and manual responses to technical queries, broadening the range of disease diagnosis and treatment simulations, expanding the ESP history collection database, and advancing the ESP’s artificial intelligence for inquiry processing.”

#### Theme 1: Perceived Benefits of Systematic Teaching

Participants noted that the digital simulation experimental teaching system for severe pelvic trauma significantly improved teaching efficiency and effectiveness, overcoming the traditional teaching constraints related to time and space.

#### Theme 2: The Appropriateness of Case Application Subjects

Most participants pointed out the variable difficulty of teaching cases within the system for different learning groups, highlighting the advanced design. The freedom for students to interact with the ESP in the system underscores its innovative development. The immersive simulation for diagnosis, treatment training, and assessment allowed students to thoroughly apply their theoretical knowledge and skills, presenting a notable challenge.

#### Theme 3: The Extendibility of Course Application

The majority of participants regarded case-based digital simulation systems as potent educational tools for both undergraduate and graduate training. A substantial number of participants viewed the system as suitable for integration into an objective structured clinical examination.

#### Theme 4: The Limitations of Systematic Research

Some limitations of the digital simulation software were reported by participants, particularly issues with the ESP not always accurately recognizing the semantics and tone of the inquiries.

#### Theme 5: Recommendations for Enhancing Systematic Research

A large case base, different training paths, and smarter ESP interaction can enhance the freshness, challenge, and realism of the ESP experience for the trainers.

### Theoretical Knowledge Level of Severe Pelvic Trauma

A comparison of theoretical examination scores before and after participants used the digital simulation software for severe pelvic trauma showed significant improvements in their overall scores for diagnosing and treating the condition, making preliminary diagnoses, the sequence of disease treatment, emergency management of hemorrhagic shock, and performing external fixation of pelvic fractures (Table 4). The IQR box plots for the theoretical knowledge levels of severe pelvic trauma, both pretest and posttest, are provided in Multimedia Appendix 4.

**Table 4:** Mean scores at presimulation and postsimulation for the 5 uncoached assignments.

Severe pelvic trauma clinical skill training	Presimulation score, % (SEM)	Postsimulation score, % (SEM)	Mean difference^a^ (95% CI)	*t* test (df=39)	Cohen *d*	*P* value^b^
Order of diagnosis and treatment	49.9 (2.0)	85.5 (1.4)	35.5 (32.7-38.3)	25.9	3.4	.001
Make a preliminary diagnosis	45.1 (1.4)	89.4 (1.0)	44.4 (42.2-46.5)	41.8	6.0	.001
Order of disease treatment	69.4 (1.8)	95.2 (0.5)	25.8 (22.2-29.5)	14.3	3.2	.001
Emergency treatment of hemorrhagic shock	39.7 (0.9)	85.8 (0.8)	46.1 (43.6-48.6)	37.6	8.5	.001
External fixation operation of pelvic fracture	32.7 (2.3)	91.1 (0.7)	58.4 (53.4-63.3)	24.1	5.3	.001

^a^The analysis included only paired data. The mean difference is the difference in mean presimulation score and mean postsimulation score.

^b^*P* value obtained from a paired 2-tailed *t* test.

## Discussion

### Principal Findings

This study yielded 3 primary findings. First, we developed a case-based digital simulation teaching system for severe pelvic trauma, incorporating principles of basic and clinical medicine. In contrast to traditional training methods, the VR system allows students to engage in repeated practice at their own pace, providing immediate and standardized feedback after each interaction. This feature overcomes challenges like high teacher-student ratios and insufficient feedback, which are common in traditional training environments. Furthermore, the use of a computer model to demonstrate physiological hemodynamic changes has been shown to aid in understanding the connection between clinical phenomena and underlying knowledge. Second, the software’s acceptability, perceived ease of use, and perceived usefulness were highly regarded by users. Finally, the application of this digital simulation teaching system resulted in a significant improvement in all participating knowledge and skill scores. These findings contribute to the innovation in severe pelvic trauma skills training and may offer guidance for the development of enhanced training strategies and the revision of orthopedic surgery training standards.

### Comparison to Prior Work

Although severe pelvic trauma is relatively rare in China, our hospital, being an orthopedic center of excellence, sees a higher incidence, treating over 100 patients annually and performing more than 20 external pelvic fixation procedures. The design of our case-based VR simulation curriculum for severe pelvic trauma draws from real cases, expert consensus, and literature reviews. Although previous research has demonstrated the efficacy of integrated learning [25], simulation training [26,27], traditional CBL [13], online learning [28], and digital patient simulator-assisted learning [29] in orthopedic clinical skills training, few studies have combined these methodologies. To our knowledge, this research is the inaugural study to amalgamate these proven effective training methods to enhance severe pelvic trauma clinical skill training, using a hybrid approach to assess the digital simulation efficacy.

Participants’ acceptance of this clinical skills training was evident in several areas. Most participants felt the simulation training provided a compelling immersion experience, was accessible at any time and location, and had clear and understandable case scenario goals. Previous studies indicate that digital simulation software can significantly impact learning success [30]. Moreover, the degree of immersion is crucial in VR software, as identification with a digital character directly influences learning motivation and effectiveness [31]. Concerning the utility, all students concurred that the self-directed exploration learning method facilitated a deeper understanding of the knowledge and skills necessary for treating severe pelvic trauma and bolstered their confidence in handling similar conditions in real-life scenarios.

However, acceptance of clinical skill training was lower in certain aspects. A minority of trainees felt that the digital simulation technology’s construction of the clinical environment and ESP allowed for experimentation within a safe psychological space. Studies suggest that digital simulators are effective for training doctor-patient communication skills [32]. Nevertheless, the discrepancy between virtual scenarios and real-life situations led to challenges in caring for actual patients. In our pilot study, most learners reported difficulty in direct communication with the ESP in the virtual environment, including eye and body language, and in discerning the nuances of the real language environment (such as tone and intonation); hence, they did not fully practice effective doctor-patient communication skills. Future educational efforts in all hospital departments should prioritize teaching doctor-patient communication skills to students.

Differences in the efficacy of case-based VR in pelvic trauma clinical skills training were observed between undergraduate and graduate students. Undergraduate respondents felt that after undergoing training with the digital simulation system, they solidified their basic medical knowledge and mastered the diagnostic and treatment processes for severe pelvic trauma; however, they expressed a lack of confidence in performing pelvic fracture external fixation. Graduate respondents believed that systematic training deepened their understanding of the diagnosis, treatment, and operational procedures for antihemorrhagic shock therapy and pelvic fracture external fixation. These variances are attributable to their respective stages of learning: undergraduates possess a stronger foundation in basic medical knowledge, while graduates have more opportunities to apply clinical knowledge in practice. Furthermore, undergraduate students outperformed graduate students in retaining disease-related knowledge due to their firmer grasp of basic medical principles. Conversely, their skills were slightly inferior to those of graduate students, a disparity linked to the latter’s greater internship experience and the number of surgical procedures conducted in the hospital. Thus, the training focus should be tailored to each need during severe pelvic trauma clinical skill training.

Quantitative outcome analyses revealed an overall improvement in pass rates at crucial assessment points posttraining with the digital simulation system, with external fixation of pelvic fractures displaying the most significant enhancement. Participants identified the realism and interactivity of the pelvic fracture model within the virtual environment as pivotal in elevating their learning experiences and assessment scores. Despite this, scores for external fixation of pelvic fractures were not the highest due to the inherent complexity and the necessity for interns to rotate through the orthopedic department to gain familiarity with patient care and the surgical technique [33]. Although the scores for diagnosing and treating the disease were the lowest presimulation, participants’ scores in these 2 categories were the highest.

Personal interviews confirmed that teaching software facilitates large-scale online student learning in terms of ease and effectiveness. Tailoring cases to different learner groups introduced high levels of order, innovation, and challenge. The experiment also addressed challenges such as prolonged real-world teaching durations, access to actual patients, and teaching environment constraints. However, 1 instructor cautioned that this innovative skill training should complement, rather than replace, traditional teaching methods, a sentiment echoed by other educational research [21,34].

### Implications

The digital simulation software for severe pelvic trauma provides undergraduates with an immersive learning experience that bridges theoretical knowledge and practical skills. For graduate students, it offers targeted preclinical training, preparing them for real-world trauma care. This approach enhances skill acquisition and promotes standardized training, potentially improving patient outcomes in severe trauma cases.

### Limitations

Limitations include the inability to directly compare the digital simulation teaching system for severe pelvic trauma with traditional teaching models. Moreover, being self-controlled, participants’ preexisting knowledge about the digital simulation system may have biased the outcomes. Although the participant count was sufficient for statistical analysis in this pilot study, the sample size remains limited.

### Conclusions

Case-based VR simulation of skill training is an effective educational approach for medical students learning about severe pelvic trauma. It presents a potentially resource-efficient approach to delivering high-quality education for both educators and learners.

## Data Availability

The datasets generated during or analyzed during this study are available from the corresponding author upon reasonable request.

## Appendix

Multimedia Appendix 1 Higher Resolution versions of Figures 3-7.

Multimedia Appendix 2 Software development and construction process of pelvic fracture model.

Multimedia Appendix 3 An interview outline of 7 open-ended questions.

Multimedia Appendix 4 Box plot with the median scores and IQR for pre- and posttest.

## Abbreviations

CBL: case-based learning
ESP: electronic standardized patient
VR: virtual reality
