# Supplementary figures and images for: Case-Based Virtual Reality Simulation for Severe Pelvic Trauma Clinical Skill Training in Medical Students: Design and Pilot Study
Source: JMIR Med Educ. 2025 Jan 17;11:e59850. doi: 10.2196/59850 (PMC11786138; doi:10.2196/59850)

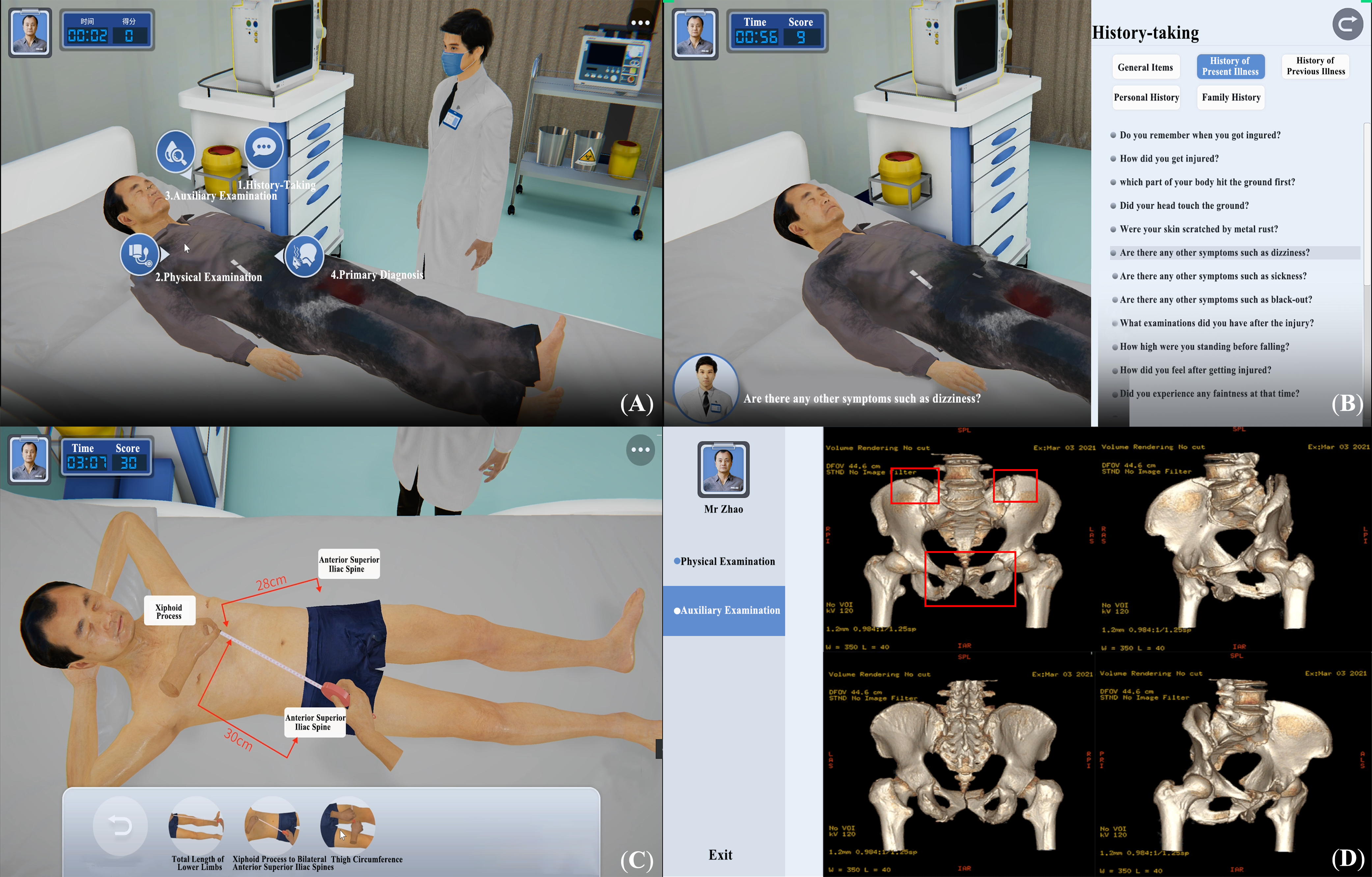


Figure 3


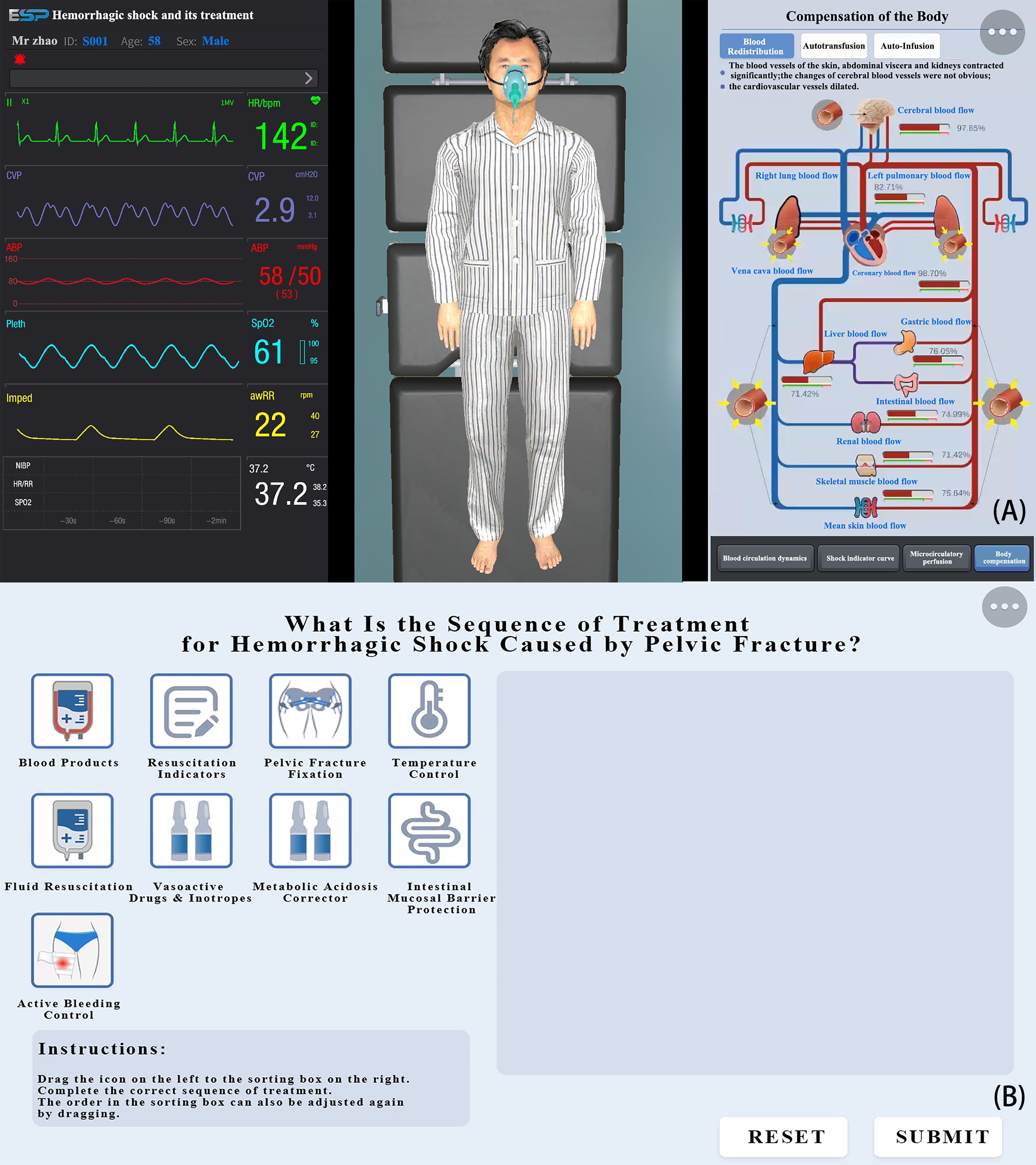


Figure 4


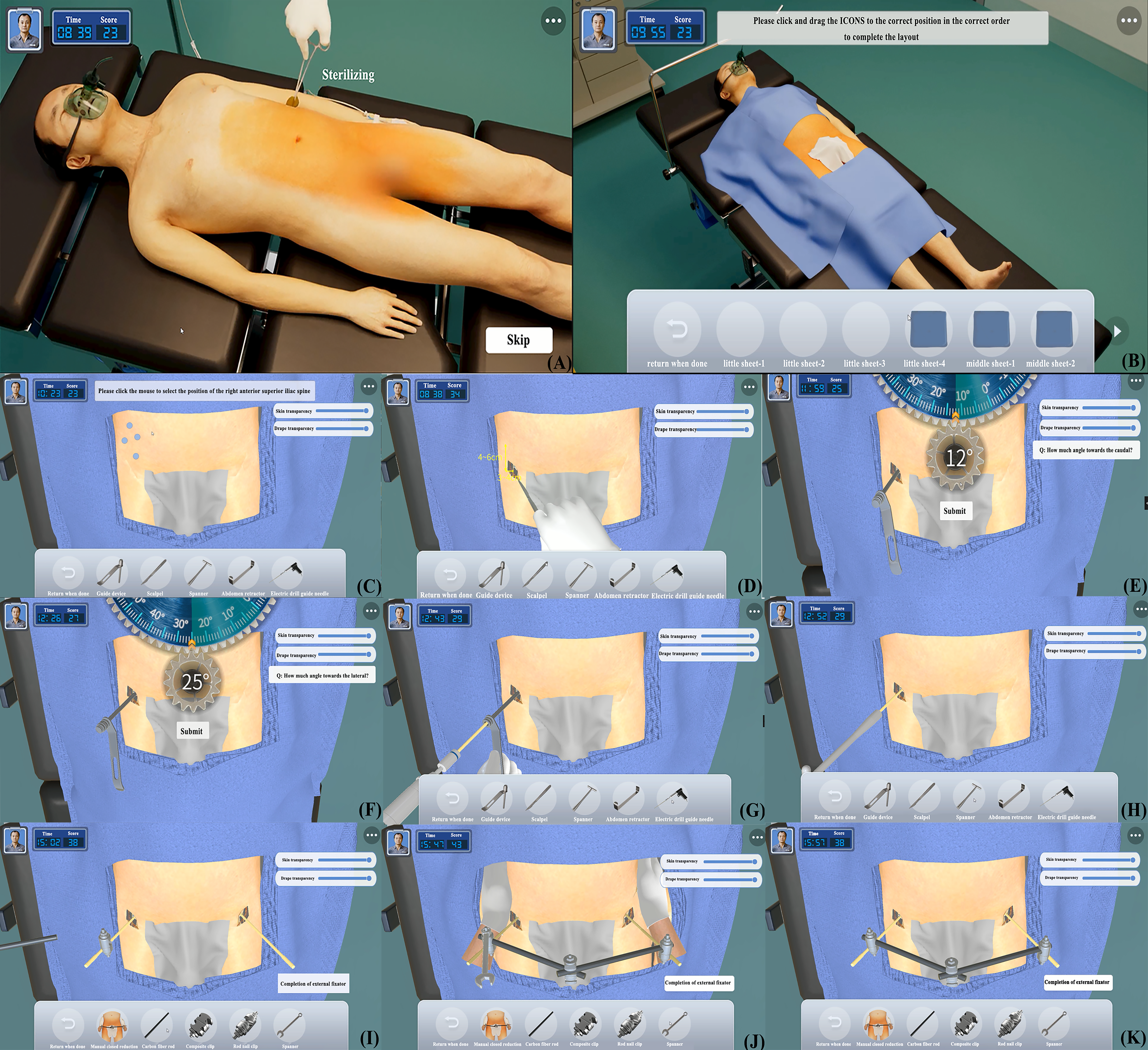


Figure 5


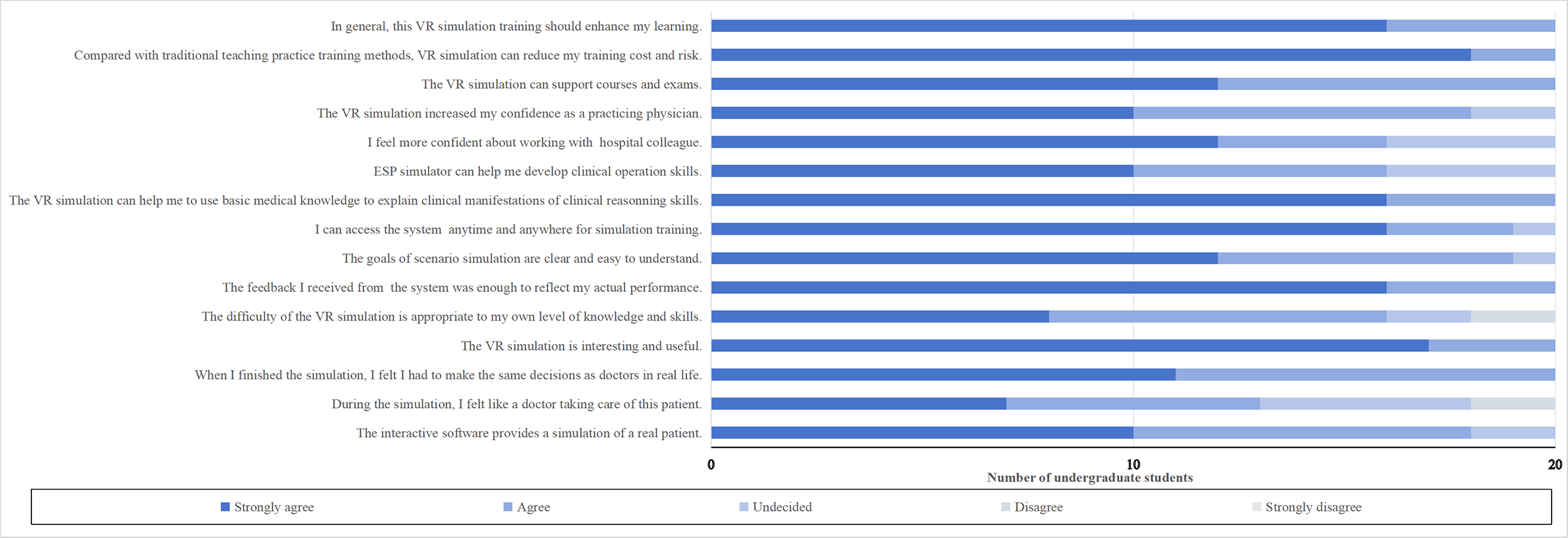


Figure 6


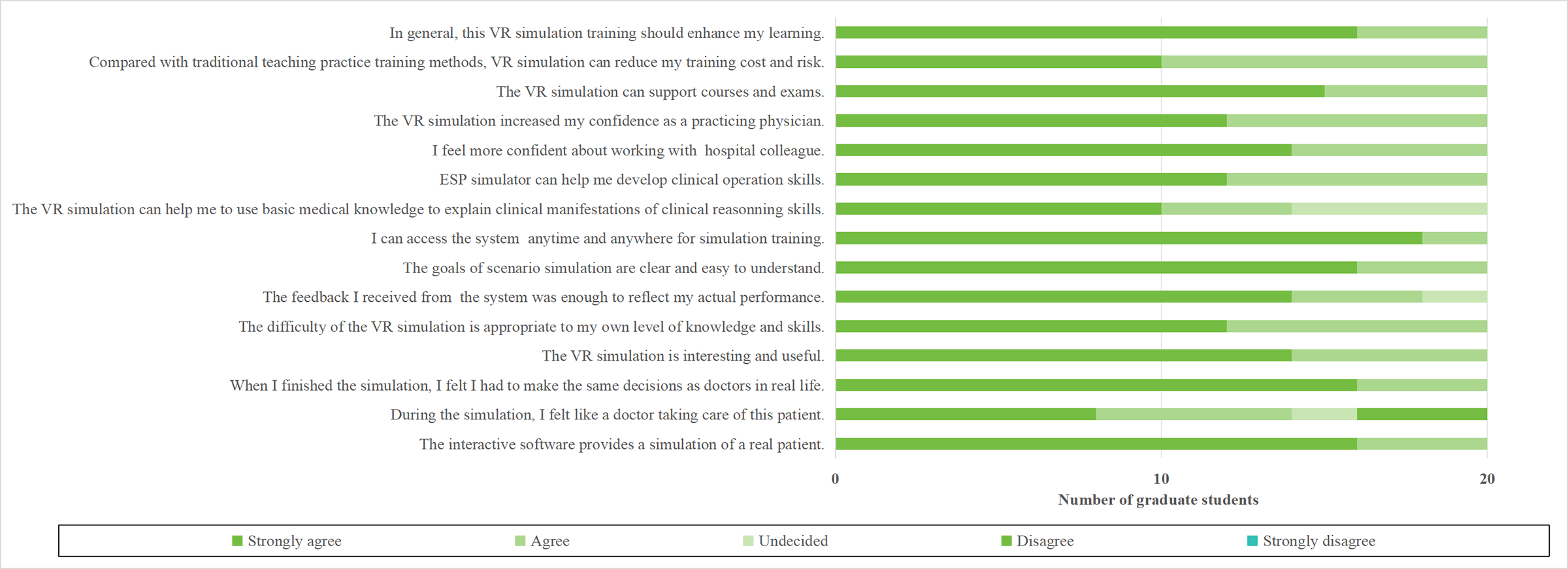


Figure 7

Supplement: Multimedia Appendix 1 [file mededu_v11i1e59850_app1.docx]
