# Supplementary material for: Case-Based Virtual Reality Simulation for Severe Pelvic Trauma Clinical Skill Training in Medical Students: Design and Pilot Study
Source: JMIR Med Educ. 2025 Jan 17;11:e59850. doi: 10.2196/59850 (PMC11786138; doi:10.2196/59850)
Supplement: Multimedia Appendix 2 [file mededu_v11i1e59850_app2.doc]

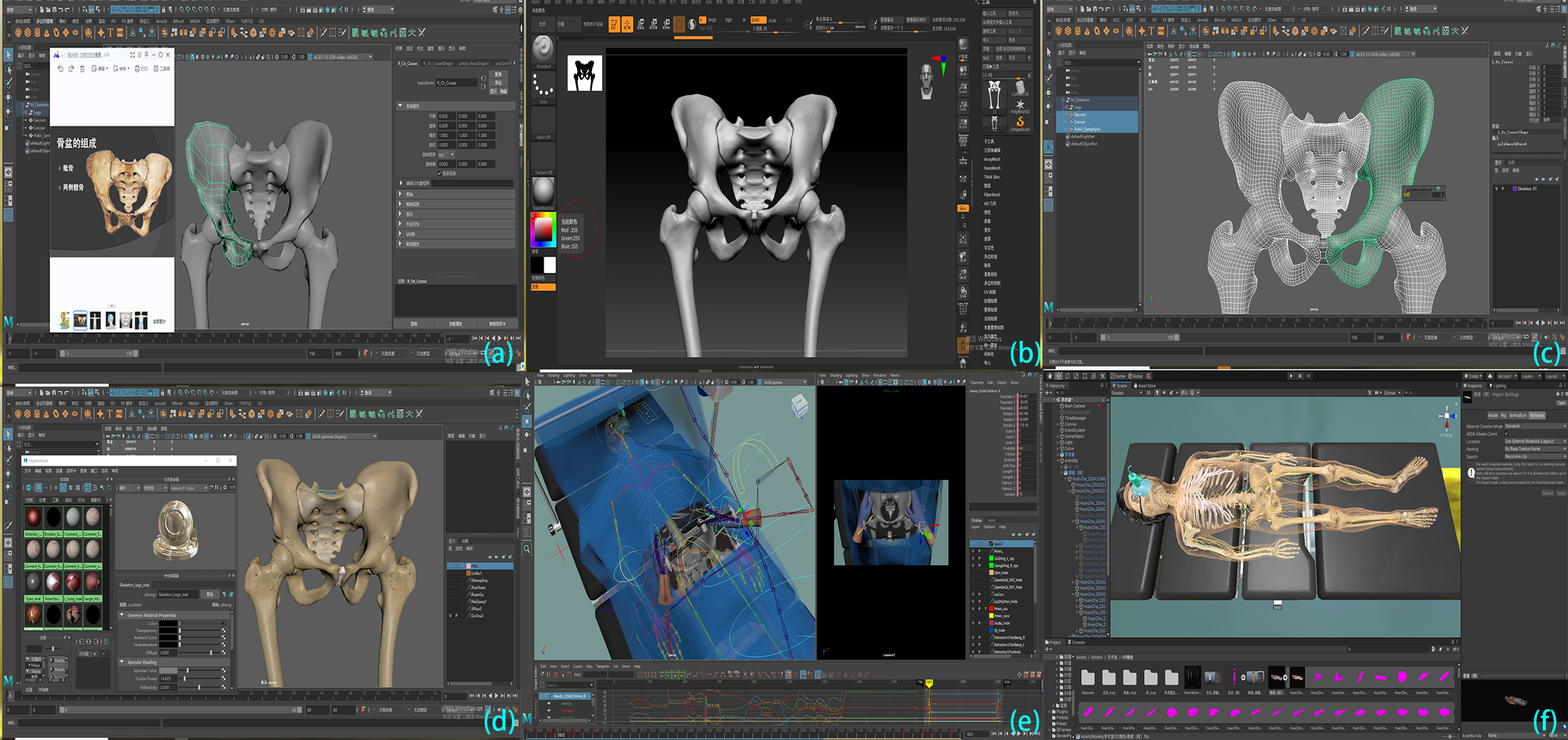
The process of establishing pelvic fracture model. Firstly, 3D modelers created a pelvic base model in the Maya development tool (a). Secondly, they used the ZBrush development tool to sculp and refine the pelvic fracture base model (b). Then, 3D modelers applied Maya's support model subdivision level adjustment function to increase the subdivision level of the pelvic fracture model (c). Next, materials and textures were added to the pelvic fracture model in Maya development software (d). Fifth, a skeletal system was established and bound to the pelvic fracture model in Maya software by 3D binding animators (e). Finally, the Unity technical art staff exported the pelvic fracture model in Maya to Unity, while Unity programmer provided real-time interaction and real-time rendering of pelvic fracture models(f).
