# Supplementary material for: Case-Based Virtual Reality Simulation for Severe Pelvic Trauma Clinical Skill Training in Medical Students: Design and Pilot Study
Source: JMIR Med Educ. 2025 Jan 17;11:e59850. doi: 10.2196/59850 (PMC11786138; doi:10.2196/59850)
Supplement: Multimedia Appendix 3 [file mededu_v11i1e59850_app3.doc]

Interview outline

| Number | Open Questions |
| --- | --- |
|
| 1 | Does this virtual simulation experiment teaching course break through the time and space constraints and how does it affect the teaching operation and management? |
| 2 | What do you think of the appropriateness of the cases and operation procedures set up by the system? For example: Is the difficulty and innovation of the experiment really consistent with the students' existing knowledge base? |
| 3 | Would you recommend this to your peers or for other courses in teaching and will it be used among international students? |
| 4 | Do you recommend it for OSCE assessment? |
| 5 | What limitations do you think the skill training designed in this study has in its implementation? How can it be further improved? |
| 6 | What do you think of the role of skill training designed in this study in teaching and assessment? For example: What is the teaching effect and the feasibility of teaching operation? |
| 7 | Is there anything else you would like to add about the skill training designed in this study? |
