# Supplementary material for: Case-Based Virtual Reality Simulation for Severe Pelvic Trauma Clinical Skill Training in Medical Students: Design and Pilot Study
Source: JMIR Med Educ. 2025 Jan 17;11:e59850. doi: 10.2196/59850 (PMC11786138; doi:10.2196/59850)
Supplement: Multimedia Appendix 4 [file mededu_v11i1e59850_app4.doc]

A box chart of the median scores and IQR before and after the test are provided in detail below.

Figure S1. IQR box plot for theoretical knowledge level of severe pelvic trauma pre-test


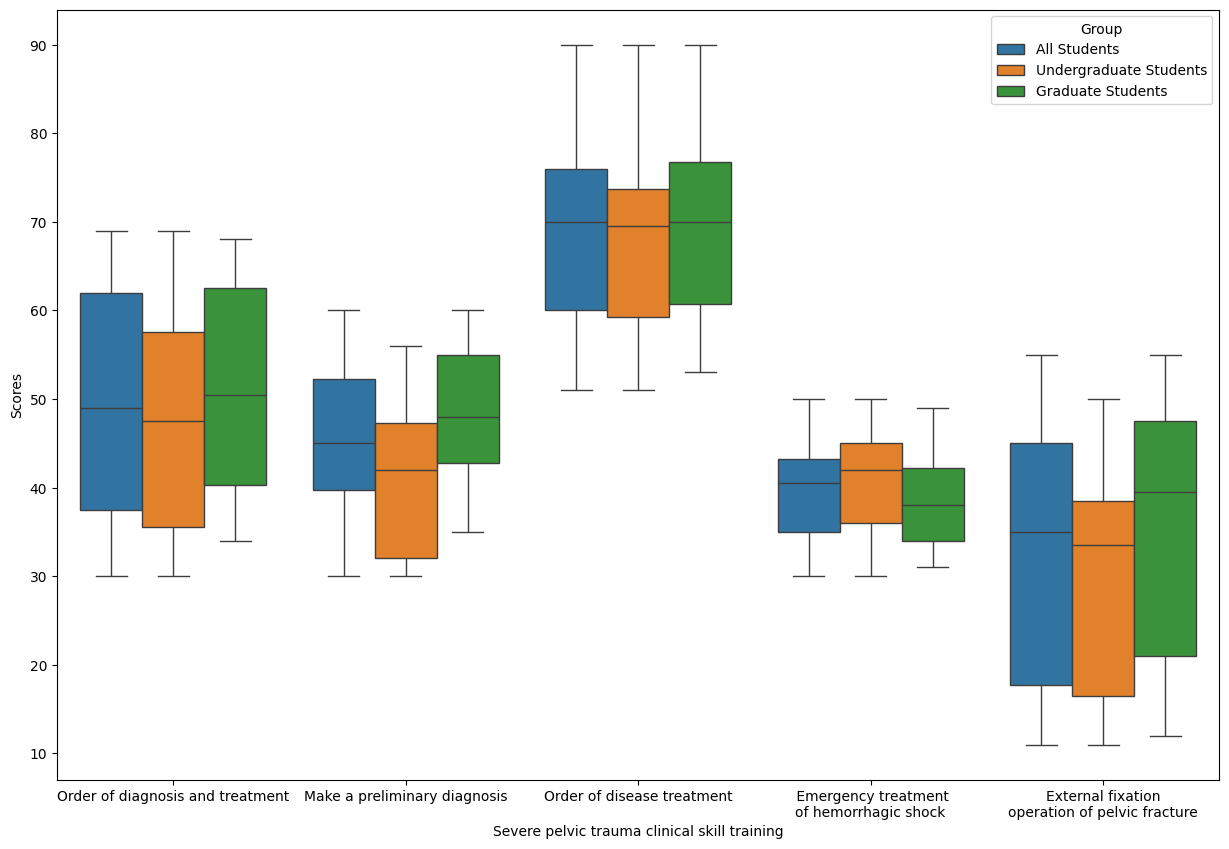


Figure S2. IQR box plot for theoretical knowledge level of severe pelvic trauma post-test


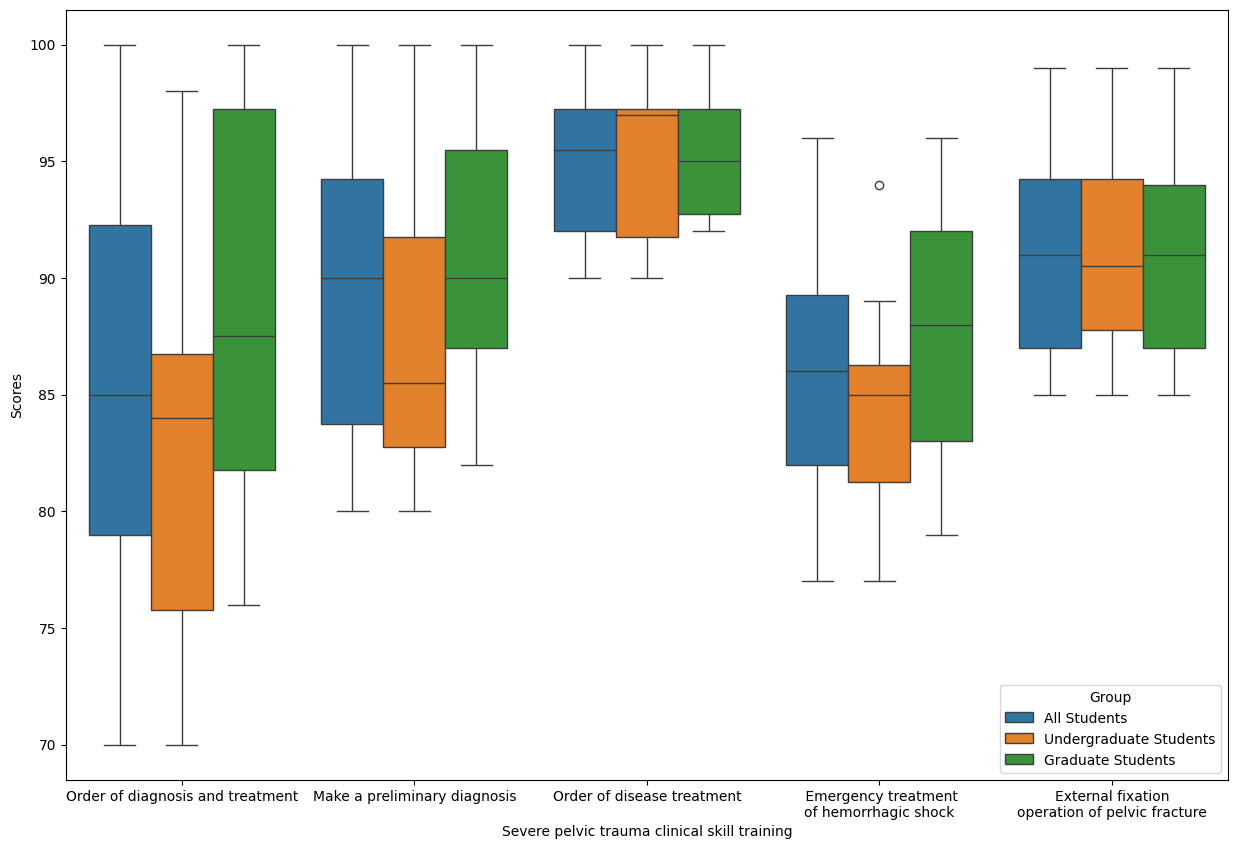


Table S1. Median scores of “Order of diagnosis and treatment” for pre-test and post-test

| Research Subject | Order of diagnosis and treatment | |
| --- | --- | --- |
|  | Median scores of pre-test | Median scores of post-test |
| All students | 49 | 85 |
| Undergraduate students | 47.5 | 84 |
| Graduate students | 50.5 | 87.5 |

Table S2. Median scores of “Make a preliminary diagnosis” for pre-test and post-test

| Research Subject | Make a preliminary diagnosis | |
| --- | --- | --- |
|  | Median scores of pre-test | Median scores of post-test |
| All students | 45 | 90 |
| Undergraduate students | 42 | 85.5 |
| Graduate students | 48 | 90 |

Table S3. Median scores of “Order of disease treatment” for pre-test and post-test

| Research Subject | Order of disease treatment | |
| --- | --- | --- |
|  | Median scores of pre-test | Median scores of post-test |
| All students | 70 | 95.5 |
| Undergraduate students | 69.5 | 97 |
| Graduate students | 70 | 95 |

Table S4. Median scores of “Emergency treatment of hemorrhagic shock” for pre-test and post-test

| Research Subject | Emergency treatment of hemorrhagic shock | |
| --- | --- | --- |
|  | Median scores of pre-test | Median scores of post-test |
| All students | 40.5 | 86 |
| Undergraduate students | 42 | 85 |
| Graduate students | 38 | 88 |

Table S5. Median scores of “External fixation operation of pelvic fracture” for pre-test and post-test

| Research Subject | External fixation operation of pelvic fracture | |
| --- | --- | --- |
|  | Median scores of pre-test | Median scores of post-test |
| All students | 35 | 91 |
| Undergraduate students | 33.5 | 90.5 |
| Graduate students | 39.5 | 91 |
